# Sex-Specific Differences in Post-Load Insulin Dynamics Are Independent of BMI-Based Adiposity and BIA-Derived Body Composition and Pubertal Stage in Adolescents with Obesity

**DOI:** 10.3390/jcm15093248

**Published:** 2026-04-24

**Authors:** Anelise Sonza, Aline Faquin, Graziano Grugni, Adele Bondesan, Diana Caroli, Laura Abbruzzese, Alessandro Sartorio

**Affiliations:** 1 Laboratório de Desenvolvimento e Controle Postural (LADESCOP), Centro de Ciencias da Saude e do Esporte (UDESC/CEFID), Universidade do Estado de Santa Catarina, Florianópolis 88080-350, SC, Brazil; aline.faquin@udesc.br; 2 Experimental Laboratory for Auxo-Endocrinological Research, Istituto Auxologico Italiano, Istituto di Ricovero e Cura a Carattere Scientifico (IRCCS), 28824 Piancavallo-Verbania, Italy; a.bondesan@auxologico.it (A.B.); d.caroli@auxologico.it (D.C.); sartorio@auxologico.it (A.S.); 3 Division of Auxology, Istituto Auxologico Italiano, Istituto di Ricovero e Cura a Carattere Scientifico (IRCCS), 28824 Piancavallo-Verbania, Italy; g.grugni@auxologico.it (G.G.); l.abbruzzese@auxologico.it (L.A.)

**Keywords:** beta cell function, obesity, adolescents, maturity, insulin sensitivity

## Abstract

**Background**: Sex-related differences in insulin sensitivity during adolescence remain incompletely understood, particularly in the context of obesity. Whether these differences reflect variations in basal insulin resistance or dynamic insulin responses remains unclear. **Objective**: To investigate sex differences in glucose and insulin responses during the oral glucose tolerance test (OGTT) and to explore mechanisms underlying potential dissociation between glycemic and insulinemic profiles. **Methods**: A cross-sectional analysis of 753 adolescents with obesity who underwent a standard oral glucose tolerance test (OGTT). Plasma glucose and insulin were measured at fasting and at 30, 60, 90, and 120 min. Mixed-effects models were used to examine glucose and insulin trajectories over time, including sex-by-time interactions, and to adjust for body mass index standard deviation score (BMI_SDS), pubertal stage (Tanner), metabolic syndrome (MetS), and body composition (resistance index). Multiple linear regression models were fitted to assess associations of sex with HOMA-IR, HOMA-β, total area under the curve (AUC), and phase-specific insulin AUCs. **Results**: Glucose trajectories during OGTT were similar between sexes, with no significant sex or sex-by-time interaction effects after adjustment. In contrast, insulin trajectories differed significantly by sex (sex-by-time interaction β = −0.10, *p* < 0.001). Boys exhibited higher baseline insulin levels and greater total insulin exposure (β = −11.2, *p* < 0.001), independent of BMI_SDS, pubertal stage, MetS, and body composition. Sex differences were sustained across all OGTT phases. HOMA-IR did not differ by sex, whereas HOMA-β showed a sex-related difference. BMI was positively associated with both basal and dynamic insulin measures. **Conclusions**: In adolescents with obesity, sex differences are characterized by altered dynamic insulin responses rather than differences in glycemic control. Boys exhibit greater compensatory insulin exposure during glucose challenge, independent of BMI-based adiposity, BIA-derived body composition and pubertal development.

## 1. Introduction

The period of adolescence represents a critical window for the development of insulin resistance [1,2], particularly in individuals with obesity [3,4]. A recent systematic review and meta-analysis that included 2033 studies from 154 different countries indicated that 1 in 5 children or adolescents experienced excess weight and that rates of excess weight varied by regional income and Human Development Index [5], which indicates an emerging worldwide health concern.

Pubertal hormonal changes, alterations in body composition, and sex-specific metabolic adaptations may influence glucose homeostasis [1,6]. Also, environmental factors such as diet and lifestyle, genetic predisposition, ancestry, ethnicity, physical activity, and others are key factors in the development and progression of metabolic diseases during childhood and adolescence [3,5].

Previous investigations reported sex-related differences in insulin sensitivity during adolescence [6,7]; however, such differences have not been consistently observed in adult populations [8], where variations in body composition appear to exert a greater influence. In younger individuals, particularly regarding dynamic metabolic responses assessed during the oral glucose tolerance test (OGTT), the existing body of evidence remains inconclusive and heterogeneous [7,9].

Sex-related differences in fat distribution become evident during puberty, with females tending to accumulate greater amounts of subcutaneous and peripheral adipose tissue, whereas males exhibit a higher deposition of visceral fat [7,8]. Visceral adiposity has been shown to have a stronger association with insulin resistance compared with overall adiposity [4,9]. During mid-puberty (Tanner stage 3), insulin sensitivity transiently decreases, and this change appears to be more pronounced in females. However, these alterations are only partially accounted for by differences in fat distribution and body mass index (BMI) [6,10].

Understanding the interplay among sex, body composition, pubertal maturation, and the balance between glucose exposure and insulin secretion, especially in larger, well-characterized study populations, is essential for elucidating the pathogenesis of metabolic disorders such as type 2 diabetes and for designing targeted interventions in at-risk pediatric populations. The persistence of sex differences in insulin levels after adjusting for body composition, and the mechanisms underlying compensatory hyperinsulinemia in boys with obesity, remain central questions in pediatric endocrinology and metabolism research [11,12,13].

Therefore, this study aimed to investigate sex differences in longitudinal glucose and insulin responses during OGTT in adolescents with obesity and to explore mechanistic relationships between integrated glucose exposure and insulin secretion. The central issue is whether the observed differences in insulin levels between boys and girls persist after adjustment for body composition. Additionally, it is necessary to determine whether variations in body composition and pubertal maturation can account for the compensatory hyperinsulinemia observed in boys with obesity.

## 2. Materials and Methods

This cross-sectional analysis of retrospectively collected data was approved by the Ethics Committee No. 5, Lombardy Region, Italy (approval number 17/25; date of approval: 25 March 2025; research code: 01C506; acronym: INDEMETSPED), and performed in compliance with the ethical principles outlined in the Declaration of Helsinki. At admission to the hospital, all patients and their parents had signed a written informed consent for authorizing the anonymous use of all their clinical, anthropometric, and biochemical parameters for subsequent scientific purposes.

### 2.1. Participants

Seven hundred and fifty-three adolescents [14] with obesity (303 males and 450 females), aged 10–18 years were admitted to the present study. Eligibility criteria included a BMI standard deviation score (SDS), calculated according to the Italian age- and sex-specific reference growth charts (BMI_SDS) ≥ 2.0 [15], primary (essential) obesity, and abstinence from alcohol consumption [16]. Exclusion criteria comprised pharmacologically treated diabetes, medical conditions, and concomitant therapies known to interfere with glucose metabolism, and fasting plasma glucose levels > 99 mg/dL.

All participants were hospitalized in the Division of Auxology at the Istituto Auxologico Italiano IRCCS (Verbania–Piancavallo) to participate in a structured, three-week, multidisciplinary body weight-reduction program.

### 2.2. Procedures

#### 2.2.1. Oral Glucose Tolerance Test

Fasting venous blood samples were obtained at baseline on the second day of hospitalization after a 12 h overnight fast. Subsequently, all participants underwent a standard oral glucose tolerance test (OGTT) (1.75 g of glucose/kg body weight, up to 75 g) following an overnight fast. Plasma glucose and insulin concentrations were measured at fasting (0 min) and at 30, 60, 90, and 120 min during the OGTT.

#### 2.2.2. Laboratory Analyses

Standard enzymatic methods were used to measure plasma glucose, insulin, high-density lipoprotein cholesterol (HDL-C), and triglycerides (TGs) (Roche Diagnostics, Mannheim, Germany).

#### 2.2.3. Anthropometric Data

Body weight was measured to the nearest 0.1 kg using a calibrated electronic scale (Wunder Sa.Bi., WU150, Trezzo sull’Adda, Italy), with participants dressed only in light undergarments. Standing height was measured to the nearest 0.1 cm using a stadiometer scale (Holtain Limited, Crymych, UK) with participants positioned barefoot and maintaining their eyes directed toward the horizon. Body mass index (BMI) was subsequently calculated as weight in kilograms divided by the square of height in meters (kg/m^2^). Waist circumference was measured with the participant standing upright, at the midpoint between the lower margin of the last rib and the iliac crest, at the end of a gentle expiration, using a flexible, non-stretchable measuring tape [17].

#### 2.2.4. Blood Pressure Measurements and Body Composition

Systolic and diastolic blood pressure (BP) were measured to the nearest 2 mmHg in the supine position after 5 min of rest using a mercury sphygmomanometer (Tema Certus, Milan, Italy) with an appropriately sized cuff [18]. The average of three measurements was used for analysis.

Body composition (BIA) was assessed using a multifrequency, tetrapolar bioelectrical impedance analyzer (Human-IM Scan, DS-Medigroup, Milian, Italy), which delivered an alternating current of 800 µA at 50 kHz. The resistance index at 50 kHz (height^2^/resistance) was selected as a proxy for body composition [19] because it reflects the body’s conductive volume, which is primarily determined by total body water and, by extension, fat-free mass (FFM).

For BIA, participants remained supine for 20 min before assessment, with limbs relaxed and positioned to avoid contact between body segments [16]. To enhance measurement accuracy and reproducibility, procedural conditions affecting the validity, reliability, and precision of bioelectrical impedance analysis were carefully standardized.

#### 2.2.5. Metabolic Syndrome (MetS)

According to the International Diabetes Federation (IDF) criteria [20], the diagnosis of MetS was made when three or more of the following risk factors are present: a WC ≥ 80 cm, fasting glucose (FPG) ≥ 100 mg/dL (5.55 mmoL/L) or on drug treatment for elevated glucose, systolic blood pressure (SBP) ≥ 130 mmHg or diastolic blood pressure (DBP) ≥ 85 mmHg or on antihypertensive drug treatment in a patient with a history of hypertension, fasting TG ≥ 150 mg/dL (1.7 mmoL/L) or on drug treatment for elevated TGs, and HDL-C < 50 mg/dL (1.3 mmoL/L) or on drug treatment for reduced HDL-C.

#### 2.2.6. Derived Measures

HOMA-IR to measure insulin resistance (IR) and HOMA-β for assessing the β-cell function were calculated using fasting values [21].

$$HOMA\text{-}IR=\frac{Fasting\;glucose\;(mmol/L)\times Fasting\;Insulin\;(µU/mL)}{22.5}$$

$$HOMA\text{-}\beta =\frac{20\times Fasting\;Insulin\;(µU/mL)}{Fasting\;glucose\;(mmol/L)-3.5}$$

Total glucose and insulin exposure during the OGTT were evaluated using the area under the curve (AUC) calculated by the trapezoidal method.

### 2.3. Statistical Analysis

All analyses were performed using Jamovi (version 2.7.17) [22]. Continuous variables are presented as mean ± standard deviation. Categorical variables are presented as frequencies and percentages. Baseline characteristics of the study participants were compared between boys and girls with respect to physical and metabolic characteristics using independent samples *t*-tests for continuous variables and chi-square tests for categorical variables.

Sex differences in longitudinal glucose and insulin responses during the oral glucose tolerance test (OGTT) were assessed using linear mixed-effects models. Sex, time, and their interaction were included as fixed effects, and subject ID was specified as a random intercept to account for within-subject correlation across repeated OGTT measurements. Models were estimated using restricted maximum likelihood (REML) and adjusted for body mass index standard deviation score (BMI-SDS), Tanner stage, metabolic syndrome (MetS), and body composition assessed by the resistance index (R_index). MetS was included as a binary variable (1 = MetS; 2 = no MetS), with MetS serving as the reference category. Degrees of freedom were approximated using the Satterthwaite method.

Sex differences in longitudinal glucose and insulin responses during the oral glucose tolerance test (OGTT) were evaluated using linear mixed-effects models with sex, time, and their interaction included as fixed effects. Model convergence was verified against the optimization criteria, and all models converged without warnings.

When a significant sex × time interaction was observed, post hoc pairwise comparisons between males and females at each OGTT time point (0, 30, 60, 90, and 120 min) were conducted using the Bonferroni test to adjust for multiple comparisons. Statistical significance was set at *p* < 0.05.

To assess basal insulin resistance and β-cell function, multiple linear regression analyses were performed with HOMA-IR and HOMA-β as dependent variables. Independent variables included sex (factor), BMI_SDS, MetS, Tanner stage, and R_index.

Multiple linear regression models were constructed to assess associations between sex and total AUC, adjusting for BMI_SDS, MetS, Tanner stage, and R_index. Secondary exploratory analyses were performed to examine phase-specific insulin exposure across four OGTT intervals (0–30, 30–60, 60–90, and 90–120 min) using similar multivariable regression models.

Model assumptions were verified by inspecting residual distributions and assessing homoscedasticity. Regression coefficients (β), 95% confidence intervals (CIs), and *p*-values are reported. The coefficient of determination (R^2^) was used to assess model fit. A two-sided *p*-value < 0.05 was considered statistically significant.

Regression coefficients (β) are presented in the original units of the dependent variable (insulin, µU/mL; glucose, mg/dL), along with their corresponding 95% confidence intervals (CIs) and *p*-values.

In the linear mixed-effects models, β coefficients represent the expected change in the outcome per unit increase in the predictor. For continuous variables (e.g., BMI_SDS and R_index), β reflects the change in the outcome per one-unit increase in the predictor. For categorical variables (e.g., sex), β represents the mean difference between groups relative to the reference category.

Time was modelled as a categorical variable; therefore, the corresponding coefficients represent differences relative to the reference time point. Interaction terms (e.g., sex × time) represent differences in the change over time between groups.

## 3. Results

### 3.1. Sample Characterization

Baseline characteristics of the study participants stratified by sex are presented in Table 1.

### 3.2. Glucose and Insulin Responses During OGTT

**Figure 1:**
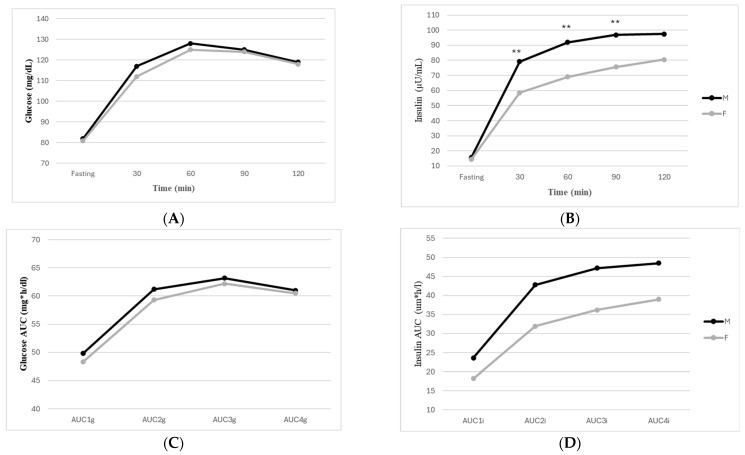
Glucose (**A**), insulin (**B**), Area Under the Curve (AUC) for Glucose (**C**), AUC for insulin (**D**) response curves during a 2 h OGTT in male (M) and female (F) groups. ** *p* < 0.01.

In the mixed-effects model, glucose levels varied significantly over time (*p* < 0.001), consistent with the expected physiological response to an oral glucose load (Figure 1A). However, neither sex nor the sex-by-time interaction was significantly associated with glucose levels in the adjusted model. Although a statistically significant sex difference was observed at fasting plasma glucose (mean difference 1 mg/dL), no meaningful differences in glucose excursion during OGTT were detected. Additionally, BMI_SDS, MetS, pubertal stage, and body composition were not independently associated with glucose trajectories.

AUCs for glucose and insulin are presented in Figure 1C,D. In line with previous findings, total glucose AUC showed only a modest association with sex (β = −5.42, 95% CI, −7.67 to −3.17, *p* = 0.04), with girls exhibiting slightly lower glucose exposure than boys (Figure 1C). However, the model explained less than 1% of the variance (R^2^ = 0.006), indicating minimal clinical relevance. BMI_SDS, pubertal stage, MetS, and body composition were not independently associated with glucose AUC.

In contrast, insulin trajectories differed significantly between sexes. A significant interaction between sex and time was observed (β = −0.10, *p* < 0.001), indicating that the insulin trajectory during the OGTT differed between boys and girls, independent of BMI_SDS, pubertal stage, MetS, and body composition (Figure 1B). At baseline, boys exhibited higher insulin levels compared to girls (β = −111.2, *p* < 0.001).

The pubertal stage was not independently associated with the insulin response in the adjusted model. In contrast, BMI_SDS, MetS, and body composition were independently associated with insulin dynamics during the OGTT. Specifically, BMI_SDS was positively associated with insulin levels (β = 6.68, *p* = 0.03), whereas greater fat-free mass, estimated by the resistance 50 kHz, was associated with lower insulin levels (β = −0.07, *p* = 0.01) and MetS (β = −8.43, *p* = 0.06) independent of sex and time.

Post hoc comparisons revealed that males exhibited consistently higher insulin concentrations at the 30, 60, and 90 min time points, with the largest mean difference observed at 60 min (mean difference ≈ 17.73 units).

**Figure 2:**
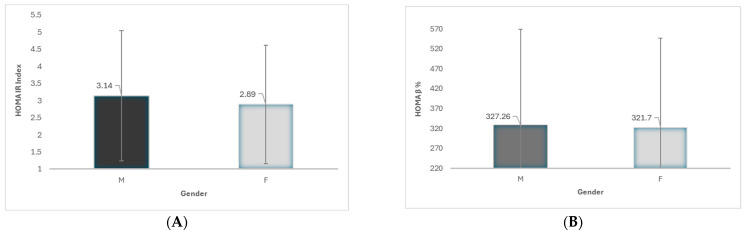
(**A**,**B**)—Homa IR and Homa β in male (M) and female (F) groups. * *p* < 0.05.

Total insulin AUC was strongly associated with sex (β = −26.16, 95% CI −38.23 to −14.10, *p* < 0.001), with girls demonstrating substantially lower insulin exposure compared to boys (Figure 2B). BMI_SDS was positively associated with insulin AUC (β = 14.95, 95% CI 2.01 to 27.89, *p* = 0.024). The resistance index was inversely associated (β = −0.11, 95% CI −0.16 to −0.07, *p* = 0.029), as well as pubertal stage (β = −5.05, 95% CI −9.14 to −0.68, *p* = 0.024) and MetS (β = −16.9, 95% CI −29.7 to −4.23, *p* = 0.009). The model explained 9% of the variance in insulin AUC (R^2^ = 0.09).

In secondary analyses examining interval-specific insulin AUC, sex remained independently associated with insulin exposure across all OGTT phases. Girls exhibited significantly lower insulin exposure compared to boys during 0–30 min (β = −4.57, 95% CI −6.61 to −2.53, *p* < 0.001), 30–60 min (β = −9.01, 95% CI −12.41 to −5.61, *p* < 0.001), 60–90 min (β = −8.44, 95% CI −12.17 to −4.89, *p* < 0.001), and 90–120 min (β = −6.51, 95% CI −10.60 to −2.42, *p* = 0.002). BMI_SDS, MetS, and body composition were also independently associated with insulin exposure in several intervals; however, the sex differences remained significant after adjustment. These findings indicate that the greater insulin exposure observed in boys was not confined to a specific phase but was sustained throughout the OGTT.

### 3.3. Baseline Insulin Resistance and β-Cell Function

In multivariable linear regression models adjusted for BMI_SDS, pubertal stage, and body composition, sex was not independently associated with HOMA-IR (R^2^ = 0.15).

However, sex was independently associated with HOMA-β (β = 31.33, 95% CI 15.47 to 47.18, *p* < 0.001; R^2^ = 0.071), with girls exhibiting higher HOMA-β values compared to boys after adjustment for obesity severity and body composition, consistent with higher estimated basal insulin secretion relative to fasting glucose. BMI_SDS was positively associated with HOMA-β (β = 65.32, *p* < 0.001), while pubertal stage, MetS, and R50 were inversely associated.

Pubertal stage was inversely associated with both indices HOMA-IR (β = −0.18, 95% CI −0.283 to −0.09, *p* < 0.001) and HOMA-β (β = −16.04, 95% CI −21.78 to −10.30, *p* < 0.001). The MetS was significantly associated with both HOMA-IR (β = −0.31, 95% CI −0.59 to −0.03, *p* = 0.026) and HOMA-β (β = −40.74, 95% CI −57.48 to −24.0, *p* < 0.001). As the coefficients reflect the contrast (No MetS—MetS), with MetS as the reference category, the negative estimates indicate that individuals with MetS had higher HOMA-IR and HOMA-β than those without MetS, consistent with increased insulin resistance and compensatory β-cell activity (Figure 2).

Taken together, while glucose regulation and fasting insulin resistance were comparable between sexes, significant differences emerged in β-cell function and dynamic insulin responses. Girls demonstrated greater basal β-cell function when obesity severity was appropriately standardized, whereas boys exhibited greater insulin exposure during the glucose challenge, independent of BMI-based adiposity, body composition and pubertal maturation.

## 4. Discussion

In this study, sex-related differences in glucose and insulin dynamics responses during the oral glucose tolerance test (OGTT) were investigated in a cohort of adolescents, after accounting for obesity, pubertal stage, metabolic syndrome (MetS), and body composition. The main findings can be summarized as follows: (a) glucose trajectories during OGTT were largely similar between boys and girls after adjustment for covariates; (b) in contrast, insulin dynamics differed substantially by sex, with boys exhibiting consistently higher insulin concentrations throughout the OGTT; and (c) girls demonstrated greater β-cell function at fasting as estimated by HOMA-β, despite similar levels of insulin resistance, after controlling variables such as BMI-based adiposity, BIA-derived body composition and pubertal stage. Together, these findings suggest that sex-related differences in glucose homeostasis during youth may be driven primarily by differences in insulin secretion dynamics rather than by differences in glycemic responses.

Glucose concentrations during OGTT followed the expected physiological pattern after oral glucose ingestion, with a marked rise followed by a progressive decline over time [23]. After adjustment for covariates, neither sex nor the sex-by-time interaction was associated with glucose trajectories, indicating that boys and girls maintained comparable glycemic regulation during the glucose challenge. Although a small difference in fasting glucose was detected, its magnitude was minimal and unlikely to be clinically significant. These findings align with previous studies demonstrating that plasma glucose levels remain tightly regulated during childhood and adolescence due to compensatory increases in insulin secretion that maintain glucose within a narrow physiological range [24,25]. Previous studies have shown that different OGTT glucose response curve patterns are associated with significant differences in insulin sensitivity and β-cell function in both adolescents [23,26] and young adults with obesity [27], despite similar fasting and 2 h glucose concentrations. These findings suggest that similar glycemic profiles can mask underlying differences in insulin dynamics and metabolic risk.

In contrast, insulin responses during the OGTT showed clear sex-related differences. Boys exhibited significantly higher insulin concentrations at multiple post-load time points, particularly during the early and intermediate phases of the test. These differences were reflected not only in mixed-effects models but also in total and interval-specific insulin AUC analyses, which demonstrated consistently greater insulin exposure in boys across all phases of the OGTT. Importantly, these associations remained significant after adjustment for BMI_SDS, pubertal stage, MetS, and body composition, indicating that sex differences in insulin dynamics are not fully explained by adiposity or developmental stage and that sex-related biological factors contribute independently to insulin regulation.

These findings are consistent with previous studies reporting sex-related differences in glucose tolerance and insulin secretion. In a large OGTT-based study, Yoshida et al. (2022) [27] observed that females exhibited higher indices of β-cell function and more favourable glucose tolerance profiles compared with males, particularly among younger individuals (22–29 years). Interestingly, the superior glucose tolerance in females was evident in young adults but attenuated in middle-aged women, which the authors attributed partly to the lower BMI observed in younger females. This finding suggests that the metabolic pattern observed in adolescence may persist into young adulthood but become less pronounced in middle age, possibly due to hormonal changes in females and age-related alterations in body composition.

The fact that boys exhibit higher insulin concentrations despite similar glucose levels suggests that greater insulin secretion—or reduced insulin clearance—may be required to maintain equivalent glycemic control. Several physiological mechanisms may contribute to this pattern. First, sex differences in peripheral insulin sensitivity may play a role [28,29]. Skeletal muscle is the primary site of postprandial glucose disposal, and variations in muscle insulin sensitivity could influence the insulin required to regulate circulating glucose [30,31]. Because muscle insulin sensitivity per unit tissue is higher in women, a given oral glucose load can often be managed with less insulin at the same glycemic level, especially before menopause [32,33]. Second, differences in hepatic insulin clearance may contribute to the observed disparities. Peripheral insulin concentrations reflect both pancreatic secretion and hepatic extraction, and alterations in hepatic insulin clearance can substantially influence circulating insulin levels [34,35]. In adults with obesity studied with tissue-specific clamps, males showed lower hepatic insulin sensitivity than females, implying relatively less effective insulin clearance [36]. In African American, Hispanic, and European American children (7–13 years), fractional hepatic extraction was significantly higher in girls than in boys (29% vs. 24%), after adjusting for age, puberty, and fat mass, whereas extrahepatic clearance showed no gender difference [37]. Third, sex-related differences in incretin hormone responses, such as glucagon-like peptide-1 (GLP-1) and glucose-dependent insulinotropic polypeptide (GIP), may modulate insulin secretion following oral glucose ingestion and, thereby, influence insulin trajectories during OGTT [38,39]. Although incretin hormones were not directly measured in our study, sex-related differences in incretin signalling could potentially contribute to the observed differences in insulin trajectories.

Emerging evidence suggests that sex-specific differences in cardiometabolic risk may originate early in life and are shaped by distinct metabolic–inflammatory pathways [40]. This study highlights that early metabolic perturbations can differentially modulate atherosclerotic processes through mechanisms such as neutrophil reprogramming, gut microbiota interactions, and maternal metabolic influences, with clear sex-dependent effects [40]. In pediatric populations, these pathways may contribute to early divergence in insulin–inflammatory regulation between boys and girls. Accordingly, the sex-related differences observed in our cohort may reflect underlying biological dimorphism in metabolic and inflammatory responses, although these mechanisms were not directly assessed in the present study.

Body composition also emerged as an important determinant of insulin dynamics. As expected, BMI_SDS was positively associated with insulin levels and insulin AUC, reinforcing the strong relationship between adiposity and compensatory hyperinsulinemia in youth. Conversely, greater fat-free mass was associated with lower insulin concentrations, suggesting that lean tissue may enhance glucose disposal and reduce insulin requirements during glucose challenge. Because skeletal muscle accounts for the majority of insulin-stimulated glucose uptake, greater lean mass may improve metabolic efficiency and thereby attenuate circulating insulin responses [30]. These results emphasize the importance of body composition beyond overall adiposity in shaping metabolic responses.

Insulin resistance is considered one of the earliest metabolic alterations associated with obesity and has been identified in more than one-third of preschool children with obesity [26]. In pediatric populations, accumulating evidence suggests that the relationship between adiposity and insulin resistance may differ according to sex [27]. Consistent with this notion, Calcaterra et al. (2021) [41] reported sex-specific associations between adiposity markers and insulin resistance parameters in children and adolescents with obesity, indicating that the metabolic impact of excess adiposity may not be uniform between boys and girls. Our findings support this concept and suggest that differences in body composition between sexes may partially contribute to the distinct insulin trajectories observed in the present study.

Interestingly, pubertal stage was not independently associated with insulin trajectories in the adjusted models. Puberty is known to be accompanied by a transient reduction in insulin sensitivity, often attributed to hormonal changes associated with growth and sexual maturation [1,6]. Sex steroids play a key role in the regulation of insulin sensitivity and secretion. Estrogens are thought to exert protective metabolic effects in females, whereas androgens in males may influence insulin dynamics and glucose metabolism. However, in the present study, the observed sex differences remained significant after adjustment for Tanner stage, suggesting that pubertal maturation alone does not fully explain these metabolic differences. Supporting this interpretation, Hammel et al. (2023) [26] reported a strong age dependency in glucose–insulin dynamics even within the same pubertal stages. In their analysis, fasting insulin and glucose levels increased before and during early puberty, remained relatively stable in mid-puberty, and subsequently declined with increasing age. These findings indicate that chronological age captures additional physiological variability beyond pubertal stage and may play an important role in shaping insulin resistance trajectories during adolescence. Taken together, these observations suggest that sex-related differences in glucose metabolism during adolescence likely reflect a complex interaction between sex hormones, age-related physiological changes, and other metabolic factors rather than pubertal stage alone.

However, in the present study, the influence of BMI-based adiposity and BIA-derived body composition appeared to outweigh the independent effect of pubertal maturation on dynamic insulin responses. This observation highlights the dominant metabolic impact of excess adiposity during adolescence and underscores the importance of considering body composition when examining metabolic changes during pubertal development.

The fasting indices provided complementary insight into sex-related metabolic regulation. Although sex was not independently associated with HOMA-IR, there is an independent association between sex and HOMA-β when adiposity was expressed as age-, sex-specific BMI_SDS, and presence of MetS. Girls exhibited significantly higher HOMA-β values after adjustment for covariates, indicating greater basal β-cell function compared with boys. This finding suggests that girls may have enhanced β-cell responsiveness, which could contribute to more efficient regulation of glucose homeostasis. Previous studies have similarly reported sex-related differences in β-cell function during adolescence [41,42,43] and young adult females [27], potentially mediated by differences in sex hormones, adipokines, or insulin clearance pathways.

The importance of assessing β-cell function in relation to insulin sensitivity has been well established in previous research. In obese youth, deterioration in this relationship—commonly described by the disposition index—represents a key early event in the progression from normal glucose tolerance to prediabetes and type 2 diabetes [44]. In this context, the higher β-cell function observed among girls in our cohort reflects a protective metabolic adaptation that helps maintain normal glucose homeostasis during adolescence.

Although HOMA-IR did not differ between sexes, this index primarily reflects hepatic insulin resistance in the fasting state. The greater insulin response observed in boys during the OGTT may indicate reduced peripheral (muscle) insulin sensitivity in postprandial conditions, which is not adequately captured by fasting indices [26].

Interestingly, despite exhibiting higher basal β-cell function, girls had lower dynamic insulin exposure during the OGTT compared to boys. This apparent dissociation between basal and post-load insulin responses suggests that sex differences in adolescents with obesity may involve complex regulatory mechanisms beyond fasting physiology and reinforce the concept that fasting surrogate indices alone may not fully capture sex-specific metabolic phenotypes during adolescence [26,41].

From a broader perspective, these findings may have implications for understanding sex differences in the early pathophysiology of hyperglycemia. Emerging evidence suggests that subtle alterations in insulin secretion dynamics during youth can precede measurable changes in glucose levels and may signal increased risk for future metabolic disease [45]. The observation that boys exhibit greater insulin exposure despite similar glucose concentrations may therefore reflect early compensatory hyperinsulinemia, a metabolic state associated with increased cardiometabolic risk later in life [23,45].

Several strengths of this study should be acknowledged, including the large sample size. The analysis incorporated detailed OGTT-derived insulin trajectories and AUC analysis, the evaluation of both dynamic insulin responses and fasting indices, and the adjustment for key metabolic and developmental factors, including obesity, pubertal stage, MetS, and body composition.

However, several limitations should also be considered. The cross-sectional design precludes causal inference, and surrogate indices of insulin resistance and β-cell function were used rather than gold-standard clamp techniques. Another weakness is the lack of body composition evaluation by Dual-Energy X-ray Absorptiometry, which would have been impractical and too expensive to perform in such a large number of subjects. Although BMI and fat-free mass were included in the models, these measures may not fully capture differences in visceral adiposity or ectopic fat distribution, which could contribute to sex-specific insulin responses. In this respect, a study from Wan et al. (2014) [19] shows that the resistance index is directly used as a predictor in regression models for FFM in adolescents with obesity, mainly if used at the group level, supporting our methodological choice. Additionally, the proportion of variance explained by some of the statistical models was relatively low (as reflected in R^2^ values), indicating that a substantial amount of variability in the metabolic outcomes remains unexplained. This is not unexpected, given the complex, multifactorial regulation of glucose metabolism and β-cell function, particularly in pediatric populations. Nevertheless, these findings should be interpreted with caution, as statistically significant associations may have limited clinical or biological relevance. Nonetheless, OGTT-derived measures remain widely used in pediatric metabolic research due to their feasibility in larger cohorts.

## 5. Conclusions

In male adolescents with obesity, insulin levels during OGTT were higher than in females, independent of BMI-based adiposity, BIA-derived body composition, pubertal stage, and MetS. These results indicate that observed sex differences in adolescents with obesity primarily reflect variation in post-load insulin dynamics rather than in fasting insulin resistance or overall glycemic control, and may be influenced by sex-specific physiological mechanisms in early metabolic regulation. Overall, these findings highlight the relevance of considering sex as a biological variable in studies of pediatric glucose metabolism and cardiometabolic risk.

## 6. Clinical Implications

The observation that boys require greater insulin secretion to maintain similar glucose levels suggests early compensatory hyperinsulinemia. Sustained hyperinsulinemia during adolescence may have long-term cardiometabolic implications independent of glycemia.

These findings underscore the importance of considering sex-specific metabolic trajectories in adolescents with obesity.

## Figures and Tables

**Table 1:** Physical and metabolic characteristics of the study group.

Variables	MALES (n = 303)		FEMALES (n = 450)		
Physical Characteristics	Mean	SD	Mean	SD	*p*
Age (aa.mm)	14.63	±2.19	14.85	±2.10	0.16
Height (m)	1.67	±0.11	1.60	±0.07	**<0.01**
Body weight (kg)	108.11	±26.42	97.06	±18.67	**<0.01**
BMI (kg/m^2^)	38.29	±6.43	37.64	±6.03	0.16
BMI_SDS	3.09	±0.62	2.98	±0.49	**<0.01**
Waist Circumference (cm)	120.09	±15.08	111.84	±13.37	**<0.01**
R_Index (ohm)	511.00	±77.60	549.00	±63.70	**<0.01**
Tanner stage (I–II/III/IV/V) n	46/36/40/76/105		43/20/42/118/227		**<0.01**
MetS (yes/no) n	105/198		101/349		**<0.01**
Metabolic characteristics	Mean	SD	Mean	SD	*p*
FPG (mg/dL)	81.90	±6.26	80.90	±6.11	**0.02**
FPI (mU/L)	15.50	±9.10	14.40	±8.28	0.08
HOMA IR	3.14	±1.9	2.89	±1.7	0.06
HOMA β	327.26	±241.50	321.70	±224.65	0.74

Legend: Data presented as mean ± standard deviation and 95%CI (LL, lower limit—UP, upper limit). Bold values represent statistical significance. Abbreviations: n, sample size; aa.mm, ages and months; m, meters; kg, kilogram; BMI_SDS, body mass index standard deviation score (BMI_SDS); BMI, body mass index; kg/m^2^, kilogram per square meter; cm, centimetres; MetS, Metabolic Syndrome; FPG, fasting plasma glucose; mg/dL, FPI, fasting plasma insulin; milligrams per deciliter; R_Index, resistance at 50 kHz.

## Data Availability

Raw data will be available upon a reasonable request to the corresponding author.
